# Efficacy and safety of Shufeng Jiedu capsule in the treatment of COVID-19: A protocol for systematic review and meta analysis

**DOI:** 10.1097/MD.0000000000023293

**Published:** 2020-12-11

**Authors:** Li Ma, Ji-Ni Song, Yan-Ping Song, Lin-Tao Zhao, Hao Chen

**Affiliations:** a Pharmacy College, Shaanxi University of Chinese Medicine, Xianyang, Shaanxi, China; b Arkansas State University, Jonesboro, Arkansas; c Shaanxi Academy of Traditional Chinese Medicine, Xi’an, Shaanxi, China.

**Keywords:** coronavirus disease 2019, meta-analysis, protocol, Shufeng Jiedu capsule, systematic review

## Abstract

**Background:**

Coronavirus disease 2019 (COVID-19) epidemic is spreading worldwide. Shufeng Jiedu capsule (SFJDC) is a commonly used drug in the treatment of COVID-19. However, there is insufficient evidence for clinical efficacy and safety.

**Methods:**

Two authors will independently search the Chinese National Knowledge Infrastructure (CNKI), VIP database, Wanfang database, the Cochrane Library, EMBASE, PubMed and Web of Science, in English and Chinese. All analysis will be performed based on the Cochrane Handbook for Systematic Reviews of Interventions. Review Manager 5.3 and Stata 16.0 software will be used to analyze the eligible data.

**Results:**

This protocol will conduct a systematic review and meta-analysis of literature listed above, and reliable outcomes about the clinical efficacy and safety of SFJDC in the treatment of COVID-19 will be obtained.

**Conclusions:**

These findings will provide guidance for clinicians and patients using SFJDC for COVID-19 treatment.

**PROSPERO Registration Number:**

CRD42020185764.

## 1 Introduction

COVID-19 was first discovered in December 2019, which strong infectivity and pathogenicity pose a great threat to public health, and cause social and economic turbulence on a global scale.^[1,2]^ A novel coronavirus is the causative agent of COVID-19 respiratory disease,^[1]^ which has significant genetic differences with the Middle East respiratory syndrome coronavirus (MERS-CoV) and severe acute respiratory syndrome coronavirus (SARS-CoV),^[3,4]^ and its homology with SARS-CoV in bats is more than 85%.^[5]^ The novel coronavirus was named severe acute respiratory syndrome coronavirus 2 (SARS-CoV-2) by the International Committee on Taxonomy of Viruses On February 11, 2020, and the World Health Organization (WHO) has named the disease COVID-19^[6]^ on the same day.

At present, novel coronavirus infection is the main source of infection, and asymptomatic infected persons may also become the source of infection.^[5,7]^ According to the results of epidemiological investigation, COVID-19 is mainly characterized by fever, fatigue, and dry cough (little or no sputum). A few patients have symptoms such as nasal obstruction, runny nose, sore throat, and diarrhea. Severe patients develop dyspnea and/or hypoxemia about 1 week after onset. Critical patients can rapidly develop into acute respiratory distress syndrome (ARDS), septic shock, metabolic acidosis that is difficult to correct, hemorrhagic and coagulative dysfunction, multiple organ dysfunction syndrome (MODS).^[8–11]^ Respiratory droplets and close interpersonal contact are the main routes of transmission.^[12]^ Novel Coronavirus has been isolated from feces and urine, transmission and exposure of aerosols caused by feces and urine should also be prevented.^[13]^ Gastrointestinal symptoms, such as vomiting and diarrhea, may also be mental illness, poor appetite and respiratory distress, which are the atypical symptoms of children and newborns.^[14,15]^

TCM has played an important role in the prevention and control of the epidemic.^[16,17]^ COVID-19 falls under the category of “plague” in Traditional Chinese Medicine, a large number of prescriptions are used for clinical treatment.^[18]^ The treatment of COVID-19 with integration of traditional Chinese and Western Medicine has obvious advantages in improving patients symptoms, delaying the progress of disease, shortening the course of disease and reducing mortality.^[16,19]^

According to the Diagnosis and Treatment Protocol for COVID-19 (Trial Version 7), COVID-19 is divided into medical observation stage, clinical treatment stage (confirmed cases) and convalescence stage according to the course of disease development. Recommend using proprietary TCM, such as SFJDC for medical observation period, but is not limited to, whenever patients with different treatment period, as long as on the basis of accurate dialectical, the appropriate card type patients, all can be used as appropriate.^[20,21]^ However, there is the insufficient evidence of efficacy and safety of SFJDC in the treatment of COVID-19. Therefore, based on the systematic analysis of the measurement data of the published randomized controlled trials, we have completed this protocol in order to obtain the reliable conclusion.

## 2 Materials and methods

This protocol for meta-analysis and systematic review followed Preferred Reporting Items for Systematic reviews and Meta-Analysis Protocols (PRISMA-P) guidelines,^[22,23]^ and has been registered in the PROSPERO database (PROSPERO ID: CRD42020185764).

### 2.1 Inclusion and exclusion criteria

#### 2.1.1 Type of study

Randomized controlled trials, whether or not blinded, language is limited in Chinese and English.

#### 2.1.2 Object of study

All patients will meet the COVID-19 diagnostic criteria,^[20]^ and clinical manifestations:

- Fever and/or dry cough, nasal obstruction, nasal discharge, sore throat, and other respiratory symptoms.
- With the fore mentioned imaging features of the COVID-19.
- In the early stage of the disease, the counts of white blood cells and the lymphocyte are normal or decreased.

If individual with epidemiological history and has any 2 of the clinical manifestations, or has no epidemiological history but 3 of the clinical manifestations, he/she should be treated as a suspected case.

Suspected cases with one of the following etiological or serological evidences as the confirmed cases:

- Positive for real-time RT-PCR detection of Novel Coronavirus nucleic acid.
- Highly viral gene homologous to the Novel Coronavirus by sequencing.
- Positive for serum Novel Coronavirus specific IgM antibody and IgG antibody; Serum Novel Coronavirus-specific IgG antibody changes from negative to positive or increases 4 times or more in convalescence than in acute phase.

#### 2.1.3 Interventions

All patients were given routine treatment, and patients in the observation group were on the basis of the treatment, SFJDC is taken orally, the dose and course of treatment is not limited. In the control group, other drugs were added on the basis of conventional treatment, or not, once a day, the dose and course of treatment were consistent with the observation group.

#### 2.1.4 Clinical measurement indexes

Nucleic acid test results, temperature stabilization time, respiratory symptoms disappear time, pulmonary rales disappeared time, the white blood count, C-Reactive Protein, the average hospitalization time, and incidence of adverse reactions. The units of each measurement should be consistent.

#### 2.1.5 Exclusion criteria

Animal experiments and retrospective analysis of SFJDC for the treatment of COVID-19. Have serious heart, brain, liver, kidney disease, or complications. Upper respiratory tract infection caused by other viruses (influenza virus, adenovirus, respiratory syncytial virus, etc.), mycoplasma pneumoniae infection. Non-infectious diseases, such as vasculitis, dermatomyositis, and organized pneumonia.

**Figure 1:**
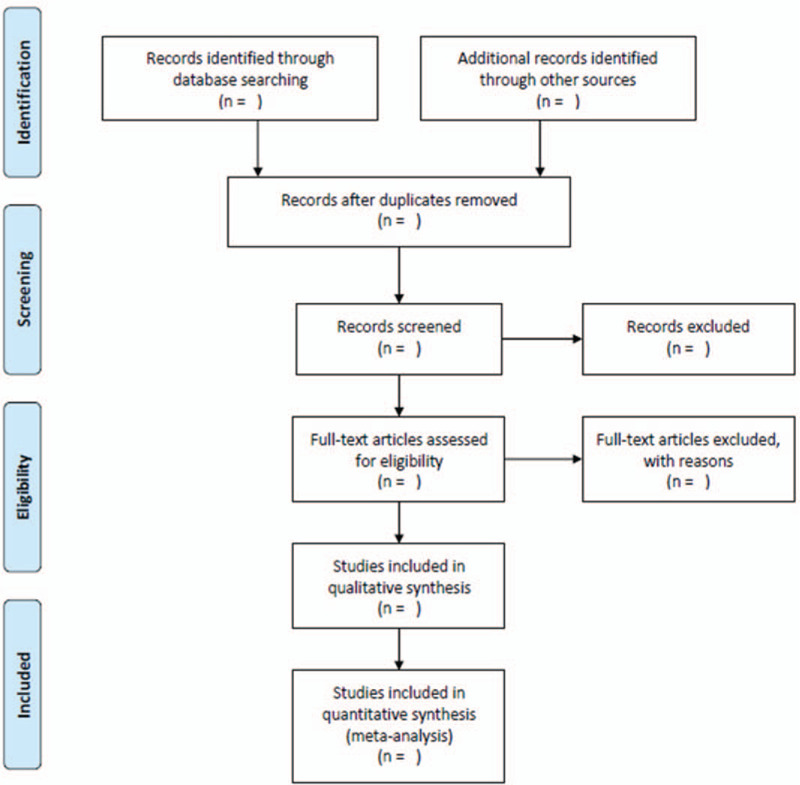
Flow chart for screening qualified studies.

Research screening process is seen in PRISMA flow chart (Fig. 1).^[23]^

### 2.2 Search strategies

The investigators will search systematically Chinese National Knowledge Infrastructure (CNKI), VIP database, Wanfang database, the Cochrane Library, EMBASE, PubMed and Web of Science in Chinese and English. Search keywords with “ Shufeng Jiedu capsules” [Title/Abstract] OR “Shufeng Jiedu ” [Title/Abstract] AND “Antiviral Agents” [Title/Abstract] OR “Antivirals” [Title/Abstract] OR “Antiviral Drugs” [Title/Abstract] OR “Drugs, Antiviral” [Title/Abstract] OR “ɑ-Interferon”[Title/Abstract] OR “Lopinavir”[Title/Abstract] OR “Ritonavir” [Title/Abstract] OR “Ribavirin” [Title/Abstract] OR “Chloroquine phosphate” [Title/Abstract] OR “Arbidol” [Title/Abstract] AND “COVID-19” [Title/Abstract] OR “2019 novel coronavirus disease” [Title/Abstract] OR “COVID-19 virus disease” [Title/Abstract] OR “2019 novel coronavirus infection” [Title/Abstract] OR “2019-nCoV infection” [Title/Abstract] OR “coronavirus disease 2019” [Title/Abstract] OR “2019-nCoV disease” [Title/Abstract] OR “COVID-19 virus infection” [Title/Abstract] AND “randomized controlled trial (RCT) [Title/Abstract] OR “randomized ” [Title/Abstract] for the search term. Two researchers independently will search and read the titles and abstracts of the literature, excluding apparently unrelated literature, reviews and pharmacological experiments. We will retrieve the full text of these articles to see if they were eligible. Disagreements will be resolved by discussion among authors.

### 2.3 Data extraction and quality evaluation

The authors will read the full text of the studies to determine whether they really meet the inclusion criteria. The content of data extraction includes the general situation of the study, the basic situation and disease situation of the 2 groups of patients, interventions and experimental outcomes. If the report is not clear, contact the author for supplement. According to the Cochrane Handbook, 7 considerations for evaluating risk of bias as follows: random sequence generation, allocation concealment, blinding of outcome assessment, blinding of participants and personnel, incomplete outcome data, selective outcome reporting, and other bias. The risks of bias will be divided into 3 levels: low-risk, high-risk, and unclear.

### 2.4 Data collection and analysis

Two searchers will independently analyze the available data from the included studies, such as sample size, intervention, and outcome measurement. A third searcher oversees the process, and if there is a disagreement, it will be resolved through discussion or negotiation.

Due to the heterogeneity of the included literature, before the statistical combination of the outcomes, the heterogeneity test should be carried out first to ensure that the differences between the results of the existing independent studies are only caused by sampling errors, otherwise, it is necessary to enter the subgroup analysis. The heterogeneity test is performed by Q statistics based on Chi-Squared test. If *I*^2^ < 50% and *P* ≥ .10 the included literature is considered to be homogeneous, and the fixed effect model is used for analysis; otherwise, it indicates that there is heterogeneity among studies, and random effect model is adopted. Generally, when considering which effect size to use, we need to consider the type of outcome indicators, when comparing between the 2 groups, if it is a continuous variable, we use weighted mean difference (WMD), standardized mean differences (SMD) to represent the effect size; if it is a binary variable, we use rate difference (RD), odds ratio (OR), relative risk (RR), and relative risk reduction (RRR) are used to express the magnitude of the effect. The results of a single experiment and the combined outcomes are described by forest plot. Sensitivity analysis is necessary, whether using different statistical models or subgroup analysis, it will help us to find possible sources of bias and correctly understand the conclusions obtained. Inverted funnel plot will be used to evaluate the potential publication bias.

The analyses will be performed using Review Manager 5.3 and STATA 16.0 software.

## 3 Discussion

Now COVID-19 is a major global challenge.^[24]^ Viral pneumonia belongs to the category of warm and hot diseases in TCM, and early intervention of TCM should be advocated in epidemic prevention and control.^[16]^ In the diagnosis and treatment protocol, it is recommended to use Chinese patent medicine such as SFJDC to relieve the symptoms of fever, fatigue and gastrointestinal discomfort.^[20]^ To some extent, it can regulate the body's immune system, avoid the outbreak of “cytokine storm”, and reduce the rate of severe infectious diseases.^[18]^SFJDC is composed of Huzhang (Polygoni Cuspidati Rhizomaet Radix), Lianqiao (Forsythiae Fructus), Banlangen (Isatidis RadixIsatis tinctoria), Chaihu (Bupleuri Radix), Baijiangcao (Herba Patrinine) Mabiancao(Verbena officinalis L.), Lugen (Phragmitis Rhizoma) and Gancao (Glycyrrhizae Radix Et Rhizoma),^[25]^ and which is often used in the treatment of acute upper respiratory tract infection. The combination of SFJDC and antiviral drugs in the treatment of COVID-19 patients can effectively control the clinical symptoms such as fever, dry cough, systemic fatigue, etc. Meanwhile, blood routine leukocyte and lymphocytes are significantly increased, chest CT absorption is obvious, and adverse reactions are less, thus shortening the hospital stay and the time of viral negative transformation.^[19,26–30]^ In this study, the writers will review the clinical evidence comprehensively and systematically to explore the efficacy and safety of SFJDC in the treatment of COVID-19. These findings will guide clinicians and patients.

## Author contributions

**Conceptualization:** Ji-Ni Song.

**Data curation:** Li Ma, Ji-Ni Song, Hao Chen.

**Investigation:** Li Ma, Ji-Ni Song, Hao Chen.

**Methodology:** Li Ma, Yan-Ping Song.

**Software:** Hao Chen.

**Supervision:** Yan-Ping Song.

**Visualization:** Ji-Ni Song, Lin-Tao Zhao.

**Writing – review & editing:** Li Ma, Yan-Ping Song, Ji-Ni Song, Lin-Tao Zhao, Hao Chen.
